# Noncoding variants and sulcal patterns in congenital heart disease: Machine learning to predict functional impact

**DOI:** 10.1016/j.isci.2024.111707

**Published:** 2024-12-28

**Authors:** Enrique Mondragon-Estrada, Jane W. Newburger, Steven R. DePalma, Martina Brueckner, John Cleveland, Wendy K. Chung, Bruce D. Gelb, Elizabeth Goldmuntz, Donald J. Hagler, Hao Huang, Patrick McQuillen, Thomas A. Miller, Ashok Panigrahy, George A. Porter, Amy E. Roberts, Caitlin K. Rollins, Mark W. Russell, Martin Tristani-Firouzi, P. Ellen Grant, Kiho Im, Sarah U. Morton

**Affiliations:** 1 Division of Newborn Medicine, Department of Pediatrics, Boston Children’s Hospital, Boston, MA, USA; 2 Fetal Neonatal Neuroimaging and Developmental Science Center, Boston Children’s Hospital, Boston, MA, USA; 3 Department of Pediatrics, Harvard Medical School, Boston, MA, USA; 4 Department of Cardiology, Boston Children’s Hospital, Boston, MA, USA; 5 Department of Genetics, Harvard Medical School, Boston, MA, USA; 6 Departments of Genetics and Pediatrics, Yale University School of Medicine, New Haven, CT, USA; 7 Departments of Surgery and Pediatrics, Keck School of Medicine, University of Southern California, Los Angeles, CA, USA; 8 Department of Pediatrics, Boston Children’s Hospital, Boston, MA, USA; 9 Mindich Child Health and Development Institute and Department of Pediatrics, Icahn School of Medicine at Mount Sinai, New York, NY, USA; 10 Division of Cardiology, Children’s Hospital of Philadelphia, Department of Pediatrics, Perelman School of Medicine, University of Pennsylvania, Philadelphia, PA, USA; 11 Center for Multimodal Imaging and Genetics, University of California San Diego, La Jolla, CA, USA; 12 Department of Radiology, School of Medicine, University of California San Diego, La Jolla, CA, USA; 13 Department of Radiology, Children’s Hospital of Philadelphia, University of Pennsylvania, Philadelphia, PA, USA; 14 Departments of Pediatrics and Neurology, University of California, San Francisco, San Francisco, CA, USA; 15 Department of Pediatrics, Primary Children’s Hospital, University of Utah, Salt Lake City, UT, USA; 16 Division of Pediatric Cardiology, Maine Medical Center, Portland, ME, USA; 17 Department of Pediatric Radiology, Children’s Hospital of Pittsburgh, University of Pittsburgh Medical Center, Pittsburgh, PA, USA; 18 Department of Pediatrics, University of Rochester Medical Center, Rochester, NY, USA; 19 Division of Genetics and Genomics, Department of Pediatrics, Boston Children’s Hospital, Boston, MA, USA; 20 Department of Neurology, Boston Children’s Hospital, Boston, MA, USA; 21 Department of Neurology, Harvard Medical School, Boston, MA, USA; 22 Department of Pediatrics, C.S. Mott Children’s Hospital, University of Michigan, Ann Arbor, MI, USA; 23 Division of Pediatric Cardiology, University of Utah School of Medicine, Salt Lake City, UT, USA; 24 Department of Radiology, Boston Children’s Hospital, Boston, MA, USA

**Keywords:** Cardiovascular medicine, Machine learning

## Abstract

Neurodevelopmental impairments associated with congenital heart disease (CHD) may arise from perturbations in brain developmental pathways, including the formation of sulcal patterns. While genetic factors contribute to sulcal features, the association of noncoding *de novo* variants (ncDNVs) with sulcal patterns in people with CHD remains poorly understood. Leveraging deep learning models, we examined the predicted impact of ncDNVs on gene regulatory signals. Predicted impact was compared between participants with CHD and a jointly called cohort without CHD. We then assessed the relationship of the predicted impact of ncDNVs with their sulcal folding patterns. ncDNVs predicted to increase H3K9me2 modification were associated with larger disruptions in right parietal sulcal patterns in the CHD cohort. Genes predicted to be regulated by these ncDNVs were enriched for functions related to neuronal development. This highlights the potential of deep learning models to generate hypotheses about the role of noncoding variants in brain development.

## Highlights

• ML predicts a subset of noncoding *de novo* variants (ncDNVs) alter gene expression
• Congenital heart disease (CHD) ncDNVs have larger predicted impacts on epigenetic marks
• Predicted gene expression changes correlate with sulcal patterns in people with CHD

## Graphical abstract

**Figure:**
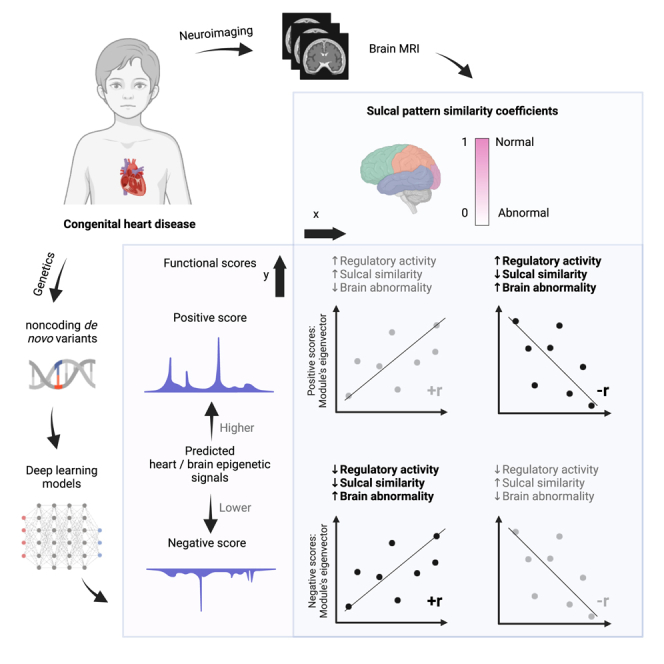

## Introduction

Sulcal patterning of the cerebral cortex develops in the fetal period, when gyrification establishes the primary pattern of cortical sulcal folds.^1^ The sulcal pattern includes the global patterns of arrangement, number, and size of primary cortical folds. These primary folds are hypothesized to form in response to early neuronal proliferation, migration and organization.^2^^,^^3^^,^^4^^,^^5^ Primary early cortical folding pattern has been shown to be influenced by genetic factors.^2^^,^^6^^,^^7^^,^^8^ Spatiotemporal characteristics of primary cortical folds are affected by the combinatorial expression pattern of multiple genes.^7^ Further supporting the role of genetics in sulcal patterning, monozygotic twins and child-mother pairs have more similar sulcal patterns than unrelated pairs.^9^^,^^10^

Sulcal pattern features can be quantified by characterization of the geometric and topological pattern of primary cortical folds from brain magnetic resonance imaging (MRI).^9^ Many cognitive disorders are thought to be due in part to alterations in the stages of brain development that also give rise to sulcal patterns.^11^ Further, as sulcal patterns remain stable into adult life,^12^^,^^13^ sulcal pattern features are strong candidate biomarkers for neurodevelopmental outcomes. For example, abnormal sulcal patterns are associated with reading performance in preschoolers/kindergarteners with a familial risk of developmental dyslexia and sulcal pattern measures predicted development of dyslexia before the appearance of clinical symptoms.^14^ In cohorts with congenital heart disease (CHD), more abnormal sulcal pattern features were correlated with greater impairments in executive function.^15^^,^^16^^,^^17^

CHD is strongly heritable, as supported by elevated chance of recurrence within families.^18^^,^^19^ A genetic etiology can be identified in approximately half of individuals with CHD, including single-gene and larger structural variants.^20^^,^^21^^,^^22^ Some forms of CHD have very high heritability such as bicuspid aortic valve and hypoplastic left heart syndrome, which have heritability estimated at 89% and 99%, respectively.^23^^,^^24^ Neurodevelopmental differences are the most common extracardiac diagnoses in people with CHD, including neurodevelopmental and structural brain differences.^25^^,^^26^^,^^27^^,^^28^ As genetic syndromes often involve both cardiac and neurodevelopmental manifestations and two-thirds of the genes with expression levels in the highest quartile of the developing mouse heart are also in the highest quartile of the developing mouse brain, genetic variants are likely to contribute to both CHD and neurodevelopmental risk.^21^ Cohort studies of participants with CHD have identified the highest prevalence of damaging *de novo* coding variants in people who also have extracardiac anomalies such as neurodevelopmental delay, consistent with genetic risk contributing to both features.^21^ Therefore, identifying genetic variants that correlate with early developmental brain features such as sulcal patterns could improve our ability to identify clinically relevant CHD variants. Despite the fact that genetic factors are known to contribute to sulcal features, the association of noncoding *de novo* variants (ncDNVs) with sulcal patterns in people with CHD remains poorly understood. The objective of this study was to determine if there is a relationship between predicted impact of ncDNVs and sulcal pattern features among participants with CHD.

Noncoding regions of the genome often have functional roles such as the regulation of gene expression. These functional regions can be identified by specific epigenetic signals such as H3K27ac modifications commonly associated with active enhancers^29^ and H3K9me2 associated with transcriptional silencing.^30^ The functional impact of each noncoding variant, such as the likelihood of disrupting an epigenetic signal that may increase or repress gene expression, can be predicted using a variety of computational approaches.^31^^,^^32^ Some models predict general noncoding variant impact, such as GERP^33^ which accounts for evolutionary constraint, and LINSIGHT,^34^ which considers molecular evolution. Successful models and frameworks such as MACIE,^35^ chromHMM^36^ and Segway,^37^ are fitted with or trained on multiple epigenetic datasets, while others such as and ChromBPNet^38^ are designed to analyze specific epigenetic data types. Machine learning (ML) models such as Basenji2^39^ and Enformer^40^ can be trained on a wide array of epigenetic datasets, and Enformer has been shown to be one of the models to best correlate with results of functional assays.^41^^,^^42^ Here, we used ML models Basenji2 and Enformer trained on heart and brain datasets to predict the functional impact of noncoding *de novo* variants (ncDNVs) on epigenetic modifications. We then identified which epigenetic modifications had larger predicted functional impact by ncDNVs from the CHD cohort compared to the non-CHD cohort. Finally, focusing on epigenetic modifications that generated functional ncDNV scores correlated with sulcal patterns, we performed gene ontology analysis of the predicted target genes to uncover mechanisms by which ncDNVs may be contributing to differences in sulcal patterns among individuals with CHD.

## Results

### CHD study group

**Figure 1:**
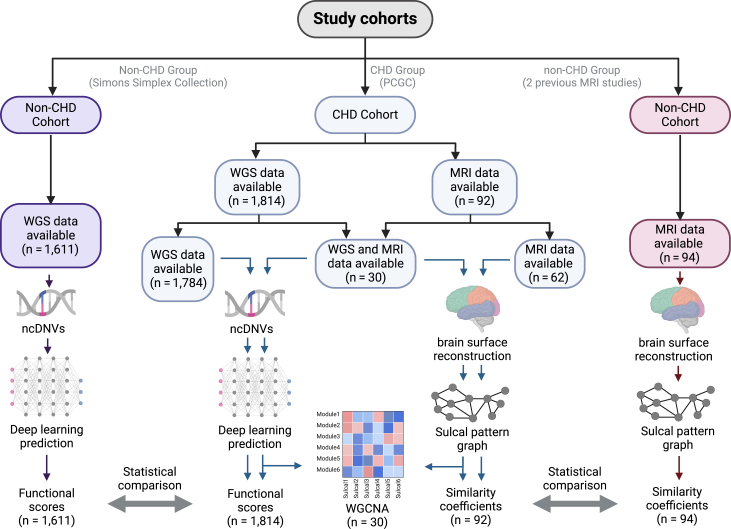
Cohort diagram depicting the number of participants in the CHD group and the non-CHD references for the different types of data and their corresponding analysis The CHD cohort consisted of 30 participants with genome sequencing data and MRI data. Additionally, 62 PCGC participants had MRI data only, while an additional 1,784 PCGC participants had genome sequencing data only. For comparing the ncDNVs with a non-CHD reference, ncDNVs of 1,611 participants that did not have a CHD diagnosis were used. A different non-CHD reference group of 94 participants was used to compare brain MRI data. Abbreviations: CHD, congenital heart disease; WGS, whole genome sequence; MRI, magnetic resonance imagining; ncDNVs, noncoding *de novo* variants.

All participants with CHD met the inclusion criteria of CHD diagnosis.^43^ Exclusion criteria included presence of a known genetic diagnosis at time of enrollment. The CHD cohort included 30 participants who had both genome sequencing data and brain MRI data (female = 11, male = 19) from the Pediatric Cardiac Genomics Consortium (PCGC; Figure 1). Individuals with a likely genetic cause of CHD identified on clinical genetic testing or research exome sequencing were excluded. An additional 62 PCGC participants had MRI data only, while an additional 1,784 PCGC participants had genome sequencing data only.

### Functional scores from general predictive model

ncDNVs were identified as previously described^20^ yielding 130,033 ncDNVs for the CHD participants with genome data, 108,919 ncDNVs for the non-CHD participants with genome data, and 2,209 ncDNVs from the 30 CHD participants with genome and brain MRI data. As a first approach, functional impact was estimated by the multivariate mixed-model framework MACIE (multidimensional annotation-class integrative estimation),^35^ which uses data from multiple epigenetic datasets to generate a prediction. Each MACIE score represents the posterior probability that the variant is either conserved or regulatory. Both conserved (median [Q1 - Q3] 3.20e-06 [7.82e-07 - 2.74e-05] CHD vs. 3.41e-06 [8.05e-07 - 2.89e-05] non-CHD; *p* = 7.43e-04) and regulatory (median [Q1 - Q3] 1.84e-11 [3.99e-12 - 9.02e-11] CHD vs. 1.95e-11 [4.24e-12 - 9.67e-11] non-CHD; *p* = 3.16e-08) scores were different between the CHD and non-CHD groups with genome data (Table S1). When subset to the highest functional score per participant, only the MACIE regulatory scores differed between cohorts (median [Q1 - Q3] 1.00 [9.99e-01 - 1.00] CHD vs. 1.00 [9.99e-01 - 1.00] non-CHD; *p* = 3.21e-02, Table S1), and the predicted target genes of those ncDNVs were enriched for functions related to hematopoiesis regulation, cytoskeleton organization, and leukocyte and myeloid differentiation (Figure S1); whereas the highest scoring variants in the non-CHD cohort were not enriched for any ontology. Therefore, as MACIE provided little insight into potential mechanisms of altered neurodevelopment, we decided to use deep learning models that are trained to predict cell- and tissue-specific impact.

### Functional scores trained on heart and brain epigenetic datasets

**Figure 2:**
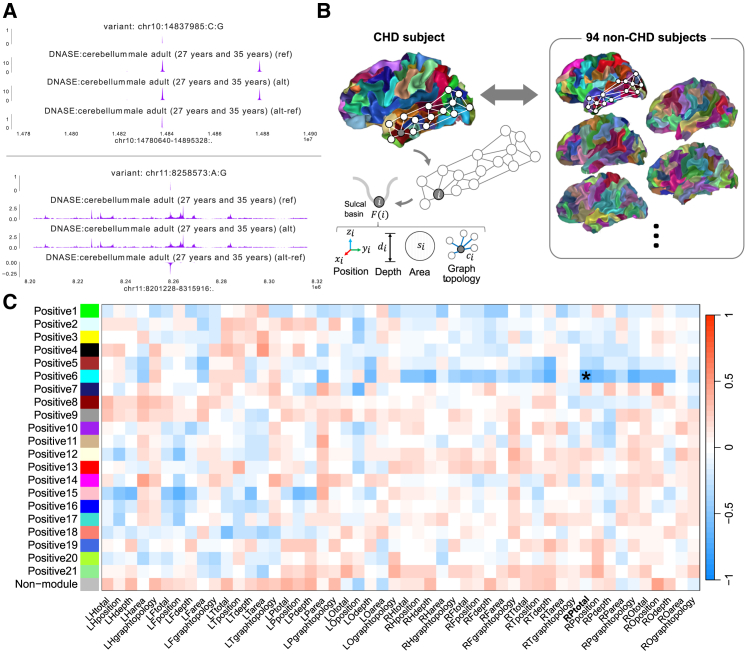
ncDNVs and sulcal pattern quantitative traits correlation in congenital heart disease (A) Sample prediction of deep learning model Enformer based on the epigenetic dataset male adult cerebellum DNase. The model predicted higher signal for the variant chr10:14837985:C:G (top) and lower signal for chr11:8258573:A:G (bottom). (B) Sulcal pattern similarity coefficients characterize the global pattern of primary sulcal folds using deep sulcal pits. Sulcal pattern graphs between different participants were automatically compared using a spectral-based matching algorithm. For every node i, the features F(i) considered were position (x_i_,y_i_,z_i_), depth (d_i_), area (s_i_), graph topology (c_i_), and all features combined (total). For every CHD participant mean similarity with a non-CHD cohort was calculated for each feature (STAR Methods). (C) Heatmap with modules-sulcal pattern coefficients correlations and Student asymptotic *p*-values of the network computed with the maximum scores. An asterisk indicates a significant correlation using Bonferroni criteria. The Bonferroni *p*-value threshold was based on the 1100 module-sulcal feature correlations (α = 0.05, *n* = 1100, *p* = 4.54e-05). Correlation and *p*-values are included in Table S5. See also Table S4. Abbreviations: CHD, congenital heart disease; L, Left; R, Right; F, frontal; H, hemisphere; O, occipital; P, parietal; T, temporal; ncDNVs, noncoding *de novo* variants.

Variants within regulatory elements could impact gene expression, for example by altering epigenetic signals. There are methods to predict the functional impact of ncDNVs derive scores from either a compendium of datasets or from a single epigenetic dataset. Such functional scores were next generated for the ncDNVs by deep learning models Basenji2^39^ and Enformer^40^ using epigenetic datasets (STAR Methods). For Basenji2 and Enformer, functional scores suggest a predicted increase in regulatory activity associated with the genomic region of interest, while negative functional scores suggest a predicted decrease in regulatory activity (Figure 2A). Of the 5,313 epigenetic datasets of which the Basenji2 and Enformer models are trained (Table S2), a subset of 583 annotations were derived from heart or brain tissues (Table S3). We predicted the functional scores for all ncDNVs from the cohort of 30 participants that had both types of data.

### Brain sulcal pattern features

Sulcal pattern features quantify the geometric and topological patterns of folds on the surface of the brain. Five sulcal pattern features (position, depth, area, graph topology, and total score) were quantified for five brain regions (frontal, temporal, parietal, occipital, and whole hemisphere) on the left and right sides, leading to 50 quantitative sulcal pattern traits (Figure 2B). We extracted the sulcal pattern features from brain MRI data using existing tools.^9^^,^^11^

### Functional impact correlation with sulcal pattern features

As the functional ncDNV scores were derived from related cell types and epigenetic modifications, we hypothesized that some functional ncDNV scores would be correlated with sulcal pattern features. Therefore, associations of sulcal pattern features with ncDNV functional scores in the CHD cohort with both ncDNV and MRI data were analyzed using Weighted Correlation Network Analysis (WGCNA). Negative and positive scores were considered separately because loss or gain of function in regulatory elements could have opposing effects on sulcal pattern features.

WGCNA with positive ncDNV functional scores contained 21 modules and a leftover non-module (Table S4). In this network, only one module, Positive6, was correlated with the right parietal total sulcal pattern similarity (r = −0.70, *p* = 2.65e-05; Table S5, Figure 2C). This association remained significant when considering age, sex, and maternal education as potential confounders (Table S6). The negative correlation value depicts how a more positive ncDNV functional score, predicted to have a greater impact on regulatory activity, corresponds to a lower sulcal pattern similarity value, which reflects a larger disruption in sulcal pattern. Forty annotations were clustered within the Positive6 module, including eight from human embryonic or induced pluripotent stem cells, and 32 from neuronal cells, including 16 cap analysis of gene expression experiments.

Next, WGCNA was repeated for negative scores. The resultant network contained 18 modules and a leftover non-module (Table S7). Two correlations of modules with sulcal features remained significant after Bonferroni correction: the Negative1 module with the right parietal graph topology sulcal similarity (r = 0.73, *p* = 8.46e-06), and the Negative1 module with the right parietal total sulcal pattern sulcal similarity (r = 0.69, *p* = 3.49e-05; Figure S2; Table S8). A more negative ncDNV functional score was correlated with lower sulcal pattern similarity score, such that a stronger loss of epigenetic regulation would correlate with a larger disruption in sulcal patterning. The Negative1 module contained 26 annotations, including five from ChIP experiments from human embryonic stem cells (HDAC6, KDM5A, H3K14ac, H3K23ac, and H3K9ac) and 21 annotations from neuronal cells, of which 14 were H3K27me3.

### Differential functional scores between cohorts

ncDNVs were identified in the remaining 1,786 participants with CHD who had genome sequencing only, yielding 127,824 ncDNVs. We also identified 108,919 ncDNVs from 1,611 participants without known congenital anomalies or neurodevelopmental diagnoses. The non-CHD genome sequencing did not overlap with the non-CHD MRI cohort. Functional scores corresponding to the CHD cohort with MRI (*n* = 30) were not different with respect to the scores from the rest of the CHD cohort (*n* = 1,784; all *p* > 2.58e-05 by two-sided Mann-Whitney *U* test, above the Bonferroni threshold *p* = 9.40e-06; Table S9).

From the 5,313 epigenetic datasets there were 985 and 191 functional scores that differed between CHD and non-CHD participants from Basenji2 and Enformer models, respectively (Bonferroni threshold *p* < 4.71e-06 to account for 10,626 comparisons; Table 1, Tables S10 and S11). Of the 583 annotations derived from heart or brain tissues, 55 and 11 functional scores were different between CHD and non-CHD cohorts by Basenji2 and Enformer models, respectively (Table 1). From both models, there were more annotations enriched for positive functional scores, than for negative functional scores in the CHD cohort. Of the 55 heart/brain annotations that differed in Basenji2 scores between cohorts, 49 were positive scores and 6 were negative scores. The data types of these annotations were chromatin immunoprecipitation (CHiP; *n* = 45), cap analysis of gene expression (CAGE; *n* = 9) and DNase chromatin accessibility (*n* = 1). Thirty-three of the positive scores were related to epigenetic modification of Histone 3 (H3), including H3K27me3 (*n* = 23), H3K4me3 (*n* = 6), H3K9me3 (*n* = 3), and H3K4me1 (*n* = 1). In the case of Enformer, nine were positive scores (five CHiP data and four CAGE) and two were negative scores (both DNase data). Eight of the positive scores were derived from neuronal tissues or cell lines (ChIP from H3K4me1, H3K9me2, H3K36me3; CAGE from pineal gland, occipital cortex and hippocampus), and one was from the left ventricle (POLR2A CHIP data). We hypothesized that ncDNV impact on these epigenetic signals could contribute to differences in brain development among people with CHD.

**Table 1:** Number of epigenetic annotations with functional ncDNV scores that differ between the CHD and non-CHD cohort for the Basenji2 or Enformer models after applying a Bonferroni *p*-value threshold of 4.71e-06

Epigenetic annotation set	Basenji2 negative scores	Basenji2 positive scores	Enformer negative scores	Enformer positive scores
All, *n* = 5,313	78	907	30	161
Heart/brain, *n* = 583	6	49	2	9

CHD, congenital heart disease.

### Sulcal pattern features differences between cohorts

**Figure 3:**
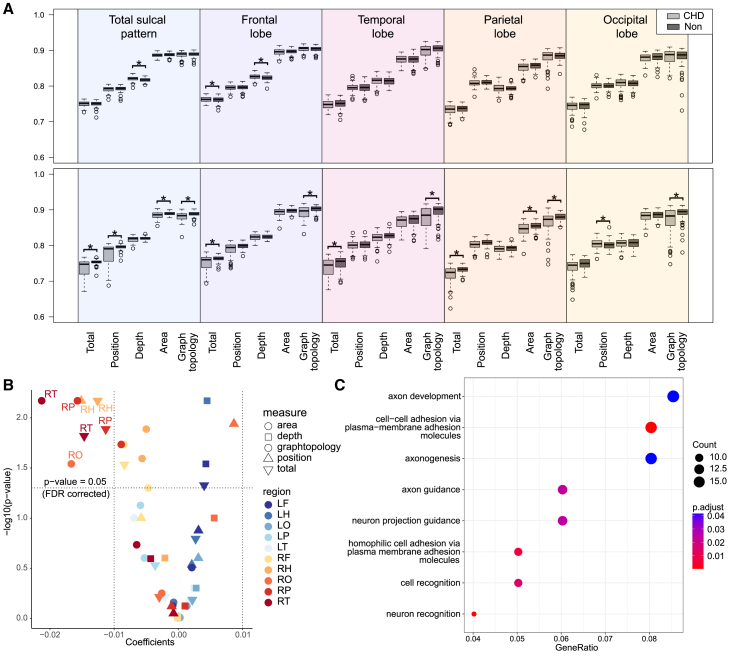
Brain sulcal pattern differences and enriched genes in close proximity to prioritized ncDNVs in CHD (A) Boxplots corresponding to brain sulcal similarity coefficients for the left (top) and right (bottom) hemispheres. Displaying the CHD (*n* = 92) (light gray) and non-CHD (*n* = 94) (dark gray) groups together in pairs. Square brackets with an asterisk depict those coefficients that were different between CHD and non-CHD (FDR-adjusted). The lower bound of the box represents the 25th percentile value, the line within the box represents the median value, and the upper bound represents the 75th percentile value. The line outside the box extends 1.5 times the interquartile range beyond the interquartile range. Values represented with a circle are greater than 1.5 times the interquartile range. All values were included in the analysis. (B) Volcano plot displaying the coefficients corresponding to CHD status (Category 0: non-CHD, Category 1: CHD status) resulted from linear regression models against the negative logarithm base 10 of their corresponding corrected FDR-adjusted *p*-value. The regression models were adjusted for magnetic field strength of the MRI scanner (1.5 T and 3.0 T). (C) Functional enrichment among predicted target genes from highest positive Enformer scores related to H3K9me2 chromatin immunoprecipitation in neural cells among the CHD participants. *p*-values were adjusted using Benjamini-Hochberg procedure. See also Table S13. Abbreviations: CHD, congenital heart disease; L, Left; R, Right; F, frontal; H, hemisphere; O, occipital; P, parietal; T, temporal; ncDNVs, noncoding *de novo* variants.

We sought to determine the degree to which the sulcal patterns of people with CHD differed from a non-CHD cohort, by measuring sulcal pattern similarity coefficients that reflect the global pattern of primary sulcal folds (Figure 3A). First, sulcal pattern features were quantified for 92 participants with CHD and 94 typically developing adolescent participants without CHD who had previously ascertained MRI data (*n* = 94).^16^^,^^17^ The 30 participants with genome sequencing data available were part of this CHD cohort. To verify that sulcal pattern coefficients of the 30 participants were not different from the rest of the CHD cohort, we compared their sulcal pattern similarity coefficients with the ones from the remaining 62 participants and found no evidence that they differ (two-sided independent t-test, FDR-adjusted; Table S12). After accounting for differences in the magnetic field strength of the MRI scanner (1.5T and 3.0T), 16 sulcal pattern features out of the 50 were different between the CHD and the non-CHD cohorts, of which 13 were from the right hemisphere (Table S13, Figures 3A and 3B). The right temporal and parietal graph topology, as well as right hemisphere position and total score, were the most altered in the CHD cohort (FDR-adjusted *p* = 0.0068 for each; Figures 3A and 3B, Table S13). The right parietal total sulcal pattern similarity, a region correlated with the heart/brain functional scores, was also different between the groups (FDR-adjusted *p* = 0.0013).

### Prioritizing correlations between ncDNV impact and sulcal features

To identify potential genetic regulation of sulcal pattern features that could be unique to CHD, we subset the WGCNA results to select correlations that also had differential functional impact or sulcal pattern features between cohorts. The H3K9me2 ChIP dataset from the neural cell annotation was included in the Positive6 module and the CHD group had higher Enformer functional scores than the non-CHD group (Table S4 and S11). Therefore, the H3K9me2-based Enformer scores were selected for further characterization. None of the Negative1 module annotations corresponded to Basenji2 or Enformer functional predictions that differed between the CHD and non-CHD cohorts so were not studied further.

### Functional enrichment in predicted target genes

To determine if there was a potential shared molecular mechanism by which ncDNVs could alter sulcal pattern, we identified the ncDNV with the highest Enformer functional score for H3K9me2 in neural progenitor cells for each CHD participant with genome sequencing data (Figure S3). Candidate target genes of the predicted regulatory regions containing each ncDNV were assigned based on linear proximity to the gene transcription start site. There were 222 genes predicted to be regulated by at least two of the 1814 ncDNVs. These genes were enriched for functions related to neuronal development and cell morphogenesis (Figure 3C; Table S14). The most enriched gene ontology term was cell-cell adhesion via plasma-membrane adhesion molecules (GO:00998742; Benjamini–Hochberg *p* = 0.00024). There were 16 cell-cell adhesion genes predicted to be regulated by CHD ncDNVs, including eight that were predicted to be regulated by at least three ncDNVs: *LRRC4C* (*n* = 5), *PCDH7* (*n* = 5), *PCDH15* (*n* = 4), *TENM4* (*n* = 4), *PCDH10* (*n* = 3), *ROBO1* (*n* = 3), *GRID2* (*n* = 3), and *KIRREL3* (*n* = 3) (Tables S14 and S15). Supporting the prediction that these ncDNVs have a regulatory role, the majority (58/89, 65%) of ncDNVs with the highest Enformer H3K9me2 in neural progenitor cells score that were predicted to regulate a gene with functional enrichment were located within a predicted transcription factor binding site from Jaspar Collection. Additionally, 14 (16%) overlapped with an ENCODE candidate Cis-Regulatory Element (cCREs) (Table S16). In the non-CHD group, there was no functional enrichment among target genes of ncDNVs with the highest Enformer functional score for H3K9me2 in neural progenitor cells.

## Discussion

This study provides an approach for exploring the association between ncDNVs and brain structure with the purpose of finding potential genetic influences on brain development in people with CHD. We identified ncDNVs in CHD participants that were predicted to have a stronger impact on the regulation of gene expression in heart and brain cells compared to ncDNVs from non-CHD participants, indicating that ncDNVs could be a mechanism underlying altered cardiac and brain development among individuals with CHD. Further, a subset of heart and brain annotations generated functional ncDNV scores that correlated with sulcal pattern traits in the CHD cohort. One functional score, trained on H3K9me2 chromatin immunoprecipitation from neural cells (H3K9me2-neural) in the Enformer model, was both higher in the CHD cohort and associated with sulcal pattern features; specifically, it was negatively correlated with right parietal total sulcal pattern similarity. The right parietal lobe has been implicated in the integration of environmental signals such as visuospatial processing and spatial attention. These are also domains that are frequently impaired among individuals with CHD.^44^ In recent studies, lower right hemisphere sulcal pattern similarity has been associated with reduced executive function in children with Tetralogy of Fallot,^16^ and with reduced executive function, general memory, and processing speed scores in larger cohort of children and adolescents with different types of CHD (single ventricle, d-transposition of the great arteries, and Tetralogy of Fallot).^45^ This could support the hypothesis that ncDNVs impact the regulation of gene expression and alter sulcal pattern formation, leading to altered neurodevelopmental outcomes. ncDNVs’ functional scores predicted with Enformer were different between cohorts in the upper functional scores. This observation suggests that an increased activity of H3K9me2, an epigenetic mark associated with transcriptional repression, might lead to a less typical development of the brain sulci. Predicted target genes of the ncDNVs in the CHD cohort were enriched for functions related to neuronal development such as cell-cell adhesion via plasma-membrane adhesion molecules and neuron recognition, which could have regional impacts due to the alteration of genes such as protocadherins with localized expression during development. Establishment of H3K9me2 signals on chromatin is important for tissue differentiation and maintenance.^30^ H3K9me2 modifications are acquired during cardiomyocyte differentiation,^46^ such that alterations in of H3K9me2 could disrupt cardiac development. A recent large-scale study has supported a difference in left-right brain traits across the lifespan. Genetic factors are one potential mechanism for these differences.^47^ Therefore, the ncDNVs prioritized in this analysis could impact both heart and brain development by increasing H3K9me2, consistent with the observation that heart and brain traits have a set of shared genetic influences.^48^

Cortical growth and folding in the human brain are associated with our capacity for high-order cognitive abilities. Sulcal patterns have been hypothesized to relate to optimal organization and arrangement of cortical functional areas and their connectivity is predetermined from genetic protomap of proliferative zones.^4^^,^^5^ During fetal development, cortical expansion occurs through the proliferation and expansion of neural stem cells and progenitors in the ventricular zone and subventricular zone.^2^^,^^6^^,^^49^ The primary pattern of sulcal folds is already noticeable during the early stage of radial growth of the cerebral cortex before the third trimester and fully determined before birth.^1^^,^^50^ Therefore, many of these changes are occurring in parallel with cardiac development, and the gradients of oxygen and trophic factors could be altered by fetal CHD. Here, the striking decrease in similarity of right sided sulcal pattern features among individuals with many types of CHD suggests that common genetic pathways are altered during fetal brain development in the setting of CHD. This could be due to germline genetic changes such as the ncDNVs studied here, or epigenetic changes induced by changes in fetal-maternal environment. Further, there may be differences in the regulation of brain-specific gene expression between hemispheres, given the stronger impact on right-sided features observed in this cohort. Alternatively, as genes that cause CHD are known to be enriched for functions related to transcriptional regulation and chromatin modification, it could be that there is a common genetic mechanism in CHD that alters neuronal expansion and radial growth independent of external changes (i.e., changes in blood flow or oxygenation). Human-specific stereotyped sulcal patterns have been hypothesized to be strongly under genetic control.^1^^,^^2^^,^^3^^,^^4^^,^^51^ Recent transcriptomic analyses of cortical germinal layers identified distinct modules or blocks of high and low gene expression that consistently mapped the location of sulcal and gyral folds in the gyrencephalic ferret and the human fetal cortex.^52^ A common cause of CHD and associated NDD is supported by shared genetic etiologies and molecular pathways between heart and brain development.^21^^,^^28^^,^^53^^,^^54^ Therefore, the genetic variants that contribute to CHD may also affect early brain development.^21^^,^^28^ For instance, some studies have demonstrated an epidemiologic and genetic overlap between CHD and NDD such as autism spectrum disorders.^55^ Additionally, genetic variants associated with brain asymmetry in CHD overlapped with autism spectrum disorder genes.^56^

Variants in brain-heart co-expressed genes are hypothesized to be an important cause of NDD in CHD. This study highlights many genes that could contribute to differences in sulcal patterning among people with CHD. *LRRC4C* and *PCDH7* had the highest number of potential regulatory ncDNVs prioritized using the H3K9me2 annotation from neural cell progenitors. Further, *PCDH7* was not the predicted target of any ncDNVs prioritized using the H3K9me2 annotation from neural cell in the non-CHD cohort. *PCDH7* has been related to central nervous system disorders^57^ and *LRRC4C* deletion variants are possible modifiers of NDD.^58^ Two other protocadherins were also predicted to be regulated by multiple ncDNVs: *PCDH15,* which is important for growth of neuronal projections and connectivity during cortical development^59^ as well as other anomalies,^60^ and *PCDH10,* which stimulates axon outgrowth and guidance.^61^ Other potential contributory genes that were prioritized using the H3K9me2-neural annotation included two associated with brain and/or heart development: *GRID2*,^62^ which is known to cause an autosomal dominant cerebellar ataxia,^63^ and *ROBO1,* which is known to be important for both axonal guidance as well as cardiac differentiation.^64^^,^^65^ A final set of genes with high-impact ncDNVs predicted using the H3K9me2-neural annotation were enriched for genes with neuronal functions. Two of those genes are also known to be related to neurodevelopmental disorders: *KIRREL3*^66^ and *TENM4*.^67^ These same genes are implicated in various stages of neurogenesis as they correlate with marker expression in single cell sequencing of human cortical cell types.^68^ NES and FABP7 are early markers of neural progenitors^69^ while DCX is a marker of later neuronal committed lineage.^70^ Expression levels of *LRRC4C*, *TENM4*, and *KIRREL3* within specific cell type clusters of the human cortex suggest that these genes play a role in early radial gliogenesis while *GRID2*, *ROBO1*, and *PCDH7* play a later role in proneural migration in DCX positive neuronal progenitors (Figure S4). The specific cell type expression suggests that these genes may participate in distinct aspects of neurogenesis. Possible future directions include assessing whether variants in these genes and others are associated with sulcal pattern differences in the general population.

This is the first study that implements deep learning models trained on heart/brain annotations to prioritize ncDNVs and assess their correlation with quantitative structural brain traits. These results suggest that noncoding variants may link to fetal brain structure in CHD. This approach highlights potential mechanisms by which ncDNVs that regulate disparate genes could impact early brain development. Similar approaches have also been applied to other congenital anomalies. A recent study of orofacial clefts was successful in training a model on 12 epigenomic features from across human development that generated variant activity difference scores that were larger for variants from affected individuals.^71^ In the future, the impact of the prioritized variants could be modeled in cellular assays for experimental validation to integrate *in silico* results with *in vitro* functional impact. Finally, such models could ultimately incorporate environmental factors to uncover the mechanisms governing the interactions between ncDNVs and brain sulcal pattern in CHD.

### Limitations of the study

There are several limitations to the current study. The non-CHD cohorts with MRI and genome sequencing data were separate, and thus we were unable to determine if the relationship between the predicted function of ncDNVs and sulcal pattern traits is different in CHD and non-CHD participants. The predicted regulatory impact of ncDNVs could be further augmented by environmental or other genetic factors which were not included in our model. Given the wide range of age of subjects in this analysis, unmeasured environmental and social determinants of health are known to impact neurodevelopment and could also influence sulcal pattern differences in our cohort. The functional scores were predicted by the models whose reliability depends on the accuracy of those models, and we did not explore every possible configuration of these two models, which could be further studied in the future. Furthermore, both heart/brain-specific models are limited to predict only for cell types available in the training dataset, as stated in the original work.^40^ With currently available data, about half of the heart and brain annotations (314/583) were from prenatal or newborn tissues or cell types. Similarly, a majority of annotations that had a larger impact among ncDNV Basenji2 scores were from prenatal or newborn tissues or cell types (30/55). By contrast, annotations that had a larger impact among ncDNV Enformer scores were predominantly from postnatal tissues or cell types (8/11). This emphasizes the need to understand how functional predictive models trained on general gene regulation may need to be adapted to developmental milieu. Future CHD studies with gene expression data available could fine-tuned the models with such data. We did not consider the impact of more than one ncDNV, as WGCNA was performed using the highest positive and negative functional scores. Another limitation is that WGCNA modules and network thresholds could determine which annotations are detected in our analysis. Additionally, from the 1,814 participants of the CHD cohort from which ncDNVs were analyzed, a modest number of 30 participants had MRI data which limits our statistical power. Larger and longitudinal cohorts representing diverse populations are also needed to determine if genetic regulation of sulcal pattern differs between CHD types. Finally, the non-CHD genetic controls did not undergo brain MRIs, precluding a direct comparison between sequenced individuals.

## Resource availability

### Lead contact

• Further information and requests for resources should be directed to and will be fulfilled by the lead contact, Sarah U. Morton (sarah.morton@childrens.harvard.edu)

### Materials availability

• This study did not generate new unique materials.

### Data and code availability

• Deidentified individual participant data (including data dictionaries), study protocols, the statistical analysis plan, and the informed consent form will be made available upon request to researchers who provide a methodologically sound proposal for use in achieving the goals of the approved proposal. Proposals should be submitted to B2BProgram@cchmc.org.
• All supporting sulcal data are provided as supplemental files, and genomic data are available to qualified investigators via dbGaP (see key resources table).
• All original code has been deposited at: https://github.com/MortonLabBCH/brain-ncdnv-chd.

## Acknowledgments

The authors would like to thank all participants and collaborators in the Pediatric Cardiac Genomics Consortium, and the ENCODE Consortium for providing epigenetic datasets. This study was funded by the 10.13039/100000097National Center for Research Resources (U01 HL098153), 10.13039/100006108National Center for Advancing Translational Sciences (UL1-TR000003, UL1-TR002541), 10.13039/100000002National Institutes of Health National Heart, Lung, and Blood Institute (U01-HL098188, U01-HL098147, U01-HL098153, U01-HL098163, U01-HL098123, U01-HL098162, and U01-HL131003, R01HL12881), NIH Centers for Mendelian Genomics (U54-HG006504), American Heart Association Career Development Award, Farb Family Fund, and Kostin Family Innovation Fund.

## Author contributions

Conceptualization, E.M.E., J.W.N., P.E.G., K.I., and S.U.M.; Data collection, S.R.D., D.J.H., H.H., and E.K.; Resources, S.R.D., E.A., M.B., J.C., W.K.C., B.D.G., E.G., D.J.H., H.H., E.K., P.M., T.A.M., A.P., G.A.P., A.E.R., M.W.R., M.T., and P.E.G.; Formal analysis and methodology, E.M.E., S.R.D., K.I., and S.U.M.; Writing – Original Draft, E.M.E., J.W.N., W.K.C., C.K.R., P.E.G., K.I., and S.U.M.; Supervision, K.I. and S.U.M.

## Declaration of interests

E.G. reported receiving grants from the NIH during the conduct of the study. M.B. reported receiving grants from the National Heart, Lung, and Blood Institute, NIH during the conduct of the study. T.A.M reported receiving grants from the NIH during the conduct of the study. P.M. reported receiving grants from the NIH during the conduct of the study. G.A.P. reported receiving grants from the University of Rochester Medical Center. A.E.R. reported receiving grants from the NIH during the conduct of the study. D.J.H. reported having a nonexclusive, royalty-free patent licensed to GEHC. J.W.N. reported receiving grant funding from the NIH, US Department of Defense, Centers for Disease Control and Prevention, Novartis, Bristol Myers Squibb, and Pfizer; being a consultant for Pfizer; and receiving honoraria from Daiichi Sankyo. C.K.R., M.T., K.I., and S.U.M. reported receiving grants from the NIH during the conduct of the study. No other disclosures were reported.

## STAR★Methods

### Key resources table

**Table:** 

REAGENT or RESOURCE	SOURCE	IDENTIFIER
**Deposited data**		

Whole genome sequencing data	Richter et al.^20^ Hoang et al.^43^	ClinicalTrials.gov: NCT01196182 dbGaP dataset # phs000571.v6.p2
Structural MRI	Morton et al.^16^ Morton et al.^17^ Morton et al.^72^	ClinicalTrials.gov: NCT01196182 dbGaP dataset # phs000571.v6.p2

**Software and algorithms**		

Basenji2	Kelley et al.^39^	https://github.com/calico/basenji
Enformer	Avsec et al.^40^	https://github.com/google-deepmind/deepmind-research/tree/master/enformer
WGCNA	Langfelder et al.^73^	https://cran.r-project.org/web/packages/WGCNA/index.html
clusterProfiler	Yu et al.^74^	https://bioconductor.org/packages/release/bioc/html/clusterProfiler.html

### Experimental model and study participant details

#### Participants

Human participants with CHD or without CHD were defined based on medical history obtained at time of enrollment. Participants with CHD (n = 1,814) were enrolled in the Pediatric Cardiac Genomics Consortium (PCGC) Congenital Heart Disease Network Study (ClinicalTrials.gov: NCT01196182) as previously described.^20^^,^^43^ This included 723 female and 1090 male participants, and one participant who did not identify a sex. At enrollment, the group had a median age of 6.5 years, interquartile age range of 2.6-11.5 years, and a range of o to 53.4 years. There were 1,448 who self-identified as white, 151 who identified as more than one race, 95 who identified as Black or African American, 88 who identified as Asian, one who identified as American Indian or Alaskan Native, and 31 who did not answer. Four-hundred and nineteen participants self-identified as Hispanic or Latino ethnicity. All participants or their parents provided informed consent and assent where applicable. Inclusion criteria included age 8 years or older and available DNA sequencing data. Individuals with a known chromosomal aneuploidy, copy number variation associated with CHD, or a likely disease-associated variant identified with research whole exome sequencing were excluded. The echocardiogram, catheterization, and operative reports were reviewed to determine cardiac phenotypes. Extracardiac structural anomalies were obtained from the medical records. For genomic analyses, a non-CHD cohort comprised of unaffected sibling participants of probands with autism spectrum disorders, who did not have CHD^20^^,^^75^ (n = 1,611), were identified in the Simons Simplex Collection. Since unaffected siblings in this quad cohort have parents’ genome data available, it was possible to extract ncDNVs.

For the sulcal pattern analysis, human participants were again defined as having CHD or not based on medical history at time of enrollment. Brain MRI data were obtained using standardized research sequences for 92 participants in the PCGC Genomic Basis of Neurodevelopmental and Brain Outcomes in Congenital Heart Disease study (ClinicalTrials.gov: NCT03070197) as previously described.^72^ This included 37 female and 55 male participants, with a median age of 15.5 years, interquartile age range of 9-22 years, and a range of 7 to 41 years. There were 85 who self-identified as white, three who identified as more than one race, two who did not answer, and one each who identified as Asian and Black or African American. Nine self-identified as Hispanic or Latino ethnicity. Among these 92, there were 30 who also had genome sequencing data. This included 11 female and 19 male participants, with a median age of 15.5 years, interquartile age range of 9-21.75 years, and a range of 8 to 38 years. There were 29 who self-identified as white and one who identified as Asian. Five self-identified as Hispanic or Latino ethnicity. Written informed consent was obtained from participants or their parents; written informed assent was obtained when appropriate. Inclusion criteria included diagnosis of CHD, age≥8 years, and available DNA sequencing data. Individuals with cardiac transplant, cardiac surgical procedure within six months of enrollment, known monogenic condition associated with abnormalities of the brain or heart, presence of pathogenic CNV, severe acquired brain injury that would overshadow the effect of a genetic variation on outcome in the opinion of the center investigator, or inability to communicate in English or Spanish were excluded. For brain MRI analyses, the non-CHD cohort data was previously ascertained from typically developing adolescents without CHD (n = 94).^16^^,^^17^ The participants w MRI cohort did not overlap with the Simons Simplex Collection participants.

#### Ethics

The study was approved by the central Institutional Review Board on December 18, 2016, and reliance agreements were approved at each study center. Written informed consent was obtained from participants or their parents; written informed assent was obtained from competent older children. Study oversight was provided by an independent data monitoring committee appointed by the National Heart, Lung, and Blood Institute.

### Method details

#### Genetic variant identification

PCR-free genome sequencing was performed as previously described.^20^ In brief, all samples were sequenced on an Illumina Hi-Seq 2000 or Illumina Hi-Seq X Ten with 150-bp paired reads to a median depth > 30x per individual. Preprocessing steps included alignment of reads to GRCh37 or GRCh38 with the Burrows-Wheeler Aligner.^76^ Genome Analysis Toolkit Best Practices recommendations were implemented for base quality score recalibration, indel realignment, and duplicate removal.^77^ Standard hard filtering parameters were used for single nucleotide and insertion/deletion variant discovery across all trios, followed by N + 1 joint genotyping and variant quality score recalibration.^78^ DNV identification was performed as previously described^20^ using three pipelines from PCGC members at Mount Sinai, Columbia and Harvard. After consolidating *de novo* calls from the three pipelines, all variants were force called with FreeBayes.^79^ DNVs with Genome Aggregation Database (gnomad.broadinstitute.org) allele frequency > 0.1% as well as DNVs in nonstandard chromosomes, segmental duplications (scores ≥ 0.99), low complexity regions, low mappability (300 bp, score < 1) regions, mucin or HLA genes, and ENCODE known problematic sites were removed.^80^^,^^81^^,^^82^

#### DNV functional estimation with mixed model framework

Multidimensional scores were obtained for each single nucleotide polymorphism DNVs using multi-dimensional annotation-class integrative estimation (MACIE).^35^ MACIE is an unsupervised multivariate mixed-model framework which was used to fit two generalized linear mixed models. The training set of those models consisted of annotations related to 1) non-synonymous coding and 2) non-coding and synonymous variants. The non-coding and synonymous coding training set consisted of 8 evolutionary conservation scores from the EIGEN database and 28 transformed epigenetic scores from the CADD database.^35^ Each MACIE score represents the posterior probability that the variant belongs to: neither conserved nor regulatory (0,0), conserved but not regulatory (1,0), regulatory but not conserved (0,1), and both conserved and regulatory (1,1). By adding these scores, three classes are obtained: MACIE conserved [(1,0) + (11)], MACIE regulatory [(0,1) + (11)] and MACIE any class [(1,0) + (0,1) + (11)]. To match MACIE’s genomic coordinates, variants were lifted using LiftoverVcf (Picard) (http://broadinstitute.github.io/picard/) from human reference genome GRCh38 (hg38) to GRCh37 (hg19). The number of variants was reduced after lifting (CHD: 125,381 ncDNVs, non-CHD: 103,701 ncDNVs). One-sided Mann-Whitney U-tests were done to compare the scores of the 3 classes between the CHD and non-CHD group using the whole set and the highest scored variant per participant for each class. Finally, functional enrichment analysis was done using the highest scored variant per participant for classes that showed difference (see STAR Methods: functional enrichment of target genes).

#### DNV functional prediction with deep learning

Functional impact predictions were generated for single nucleotide polymorphism DNVs by deep learning models Basenji2^39^ and Enformer.^40^ Different *in silico* methods are available for assessing the impact of noncoding variants using different criteria. Many of them evaluate variants based on single characteristics and are not cell- and tissue-specific. Basenji2 and Enformer, are two supervised deep learning models that predict cell-type- and tissue-specific gene expression impact using DNA sequences. As we were interested in exploring the correlation between sulcal pattern traits and ncDNVs, we needed the functional scores to be derived from heart and brain related cell types and epigenetic modifications. Both models predict regulatory activity in 128-basepair windows considering elements up to 20 kilobases (Basenji2) and 100 kilobases (Enformer) away. We employed the default test time augmentation by shifting the input sequence by a certain number of nucleotide positions forward and backward, and by getting the corresponding reverse sequence. In both cases we used the pretrained models that were trained with 5,313 epigenomic signal tracks from ENCODE,^82^ FANTOM5,^83^ and Epigenomics Roadmap,^84^ which included Cap Analysis of Gene Expression (638), DNase/Assay for Transposase-Accessible Chromatin (684), and ChIP (3,991) datasets (Table S1). Weights from pretrained models based on the 5,313 epigenetic datasets were implemented with best practices for Enformer and Basenji2, without any additional preprocessing. We did not fine-tune the parameters since we lack tissue specific gene expression data from our participants. We expect these models to generalize to our data because they are trained to predict gene expression signals from human (and mouse) DNA sequences. From those annotations, 583 related to the heart and brain were considered in this analysis, from which 414 were chromatin immunoprecipitation datasets (ChIP), 91 were Cap Analysis of Gene Expression and 78 were DNase/Assay for Transposase-Accessible Chromatin (Table S3). The models score variants by subtracting the prediction obtained when inputting a DNA sequence with the alternative allele from the prediction obtained with the reference allele.^39^ Positive high scores (> 0) would suggest that the model is predicting a stronger epigenetic signal due to the alternative allele, whereas negative high scores (< 0) would suggest weaker signal due to the alternative allele.

#### Sulcal pattern analysis

For each CHD participant, sulcal pattern was compared with each non-CHD (n=94) individual. Whereas of each non-CHD, it was compared with the remaining 93 participants. Similarity coefficients were averaged for each individual. For the CHD cohort, MRI was performed in seven different PCGC sites on either 3.0T Siemens Prisma (two sites) or Prisma Fit (three sites), or 3.0T General Electric MR750 (two sites). The parameters used were: repetition time = 2500 ms, echo time = 2.88 ms (Siemens), 2 ms (General Electric), flip angle = 8°, acquisition matrix = 256 × 256, field of view = 256 mm, and slice thickness = 1 mm.^72^ For the non-CHD cohort, MRIs were obtained with 1.5T General Electric Twin-speed magnetic resonance scanners in two centers or a 3T General Electric Twin-speed magnetic resonance scanner in one of them. The parameters used were: repetition time = 35 ms, echo time = 6 ms, flip angle = 45°, acquisition matrix = 256 × 256, field of view = 220 mm, and slice thickness = 1.5 mm.^16^^,^^17^

The images were pre-processed to extract cortical surfaces using the FreeSurfer pipeline^85^ as previously described.^17^ Once the cortical models were reconstructed, they were automatically parcellated into anatomical regions based on lobar and gyral/sulcal structure.^86^ We used the left and right whole hemispheres and lobar regions of the white matter surface (gray/white matter boundary) for sulcal pattern analysis in this study. The sulcal pattern was represented as a graph structure with sulcal pits and their surrounding catchment basins as nodes. Sulcal pits are defined as the deepest local point in a sulcal catchment basin on the cortical surface. Sulcal pits and catchment basins are substructures decomposed from one primary sulcal segment. Sulcal depth maps on the white matter surface were generated using the FreeSurfer and sulcal pits and their surrounding sulcal catchment basins were automatically identified based on a smoothed sulcal depth map using a watershed segmentation algorithm.^11^^,^^14^ If sulcal catchment basins met, sulcal pits in those basins were connected with an edge. This graph was designed to characterize the global pattern of primary sulcal folds using deep sulcal pits. Sulcal pattern graphs for participants were automatically compared to the control graph using a spectral-based matching algorithm.^9^ The sulcal pattern comparison was performed using geometric features of sulcal folds (3D position, depth, and area of sulcal pits and basins) and their inter-sulcal relationships, graph topology (the number of edges and the paths between nodes), to highlight the interrelated arrangement and patterning of sulcal folds.^9^ The optimal match was determined between two different sulcal graphs and then their similarity was computed for each hemisphere and lobar region, which ranged from 0 to 1. After measuring the total similarity with all features combined (sulcal position, area, depth, and graph topology), we further measured the similarity only using each individual feature by setting all weights of the other features to 0 to evaluate their relative importance on the sulcal pattern similarity.^9^ Using the graph-based sulcal pattern comparison method, sulcal pattern similarities of all possible pairs were automatically computed for the left and right hemispheres and lobes.

#### Weighted Correlation Network Analysis

Correlations between the sulcal pattern features and maximum or minimum functional scores from heart/brain functional annotations were performed using Weighted Correlation Network Analysis (WGCNA) via the R package *WGCNA*^73^ to generate signed hybrid networks (Figures S5A and S6A). Different networks were computed for maximum and minimum functional scores and a soft thresholding power of 9 was selected for both after applying the approximate scale-free topology criterion (Figures S5B and S6B). We performed automatic network construction and module detection^73^ by using Dynamic Hybrid tree cut’s default parameters of cut height 0.995 and a sensitivity deep split coefficient of 2 for module detection, clustering at least 20 targets in each module, and finally cutting the dendrogram at height 0.25 for merging modules whose eigengenes were highly correlated. Pearson correlations and Student asymptotic p-value were calculated between the modules’ eigengenes with the sulcal pattern coefficients. In each network, we removed one outlier based on the distance value obtained after applying hierarchical clustering to the participants’ functional scores. The sample size of 29 gave a power of 99.3% to detect correlations of the magnitude that were observed (α = 0.05, r = 0.7, n = 29). After applying Bonferroni correction, and account number of measurements and modules, we see a decrease in power to 64% (α = 4.5e-5, r = 0.7 n = 29). Additionally, to test if potential confounders influence the correlations between modules and sulcal pattern traits, we repeated significant correlations while controlling for age, sex, and maternal education level using the partial correlation.

#### Functional enrichment of target genes

The maximum and minimum functional prediction values per CHD and non-CHD participant were identified separately for each of the significant heart/brain annotations between cohorts and annotations within significantly correlated modules with sulcal pattern traits. Likely target genes were identified as those with a transcription start site in closest proximity to the ncDNV, with a maximum range of 20 kilobases. Functional enrichment was determined for annotations prioritized by the pipeline (Figure S7) using the R package *clusterProfiler*^74^ using all likely target genes of ncDNVs as background. Finally, DNA regions surrounding ncDNVs that belonged to an enriched gene from enrichment analysis were explored to find overlapping ENCODE Candidate Cis-Regulatory Elements using the UCSC Genome Browser.^87^

#### Figure preparation

Some of the figures presented in this work were created with BioRender.com with license to publish.

### Quantification and statistical analysis

#### Statistical analysis of ncDNV functional scores

To focus on ncDNVs with the highest predicted impact on gene expression, we first removed all ncDNVs with scores in the middle two quartiles of each annotation. Statistical comparisons were completed using Scipy 1.8.0 in Python 3.10.12. A one-sided Mann-Whitney *U* test was used to determine if there was a more extreme distribution of scores in the CHD vs non-CHD ncDNVs for the lower (< Q_1_) and upper (> Q_3_) quartiles. For the lower scores, the alternative hypothesis tested consisted on whether the distribution underlying CHD functional scores was stochastically less than the distribution underlying the non-CHD functionals scores, i.e. H_1,lower_:F(u) < G(u), where F(u) and G(u) are the cumulative distribution functions of the CHD and non-CHD functional scores’ distribution*s*, respectively. For the upper scores, the opposite was tested i.e. H_1,upper_:F(u) > G(u). Therefore, for each of the 5,313 annotations two tests were performed. To address multiple comparisons in genomic analyses, we applied Bonferroni correction (α = 0.05, n = 10,626, p = 4.71e-06).

#### Statistical analysis of sulcal pattern data

To compare sulcal pattern coefficients between the cohorts, we computed linear regression models for each of the 50 measurements to evaluate the extent to which CHD status (Category 0: non-CHD, Category 1: CHD status) affects sulcal measurements. Sulcal pit extraction, graph construction, and pattern analysis have been found reliable and reproducible across scan sessions, sites, and magnet strengths.^88^ However, because of potential differences between sites and scanners, we decided to follow a more stringent approach and control for magnetic field strength of the MRI scanner (1.5T and 3.0T) and guard for potential confounders.^89^ For statistical tests with sulcal pattern data where there was a small sample size, we applied FDR adjustment in R version 4.1.2, using a threshold adjusted p < 0.05 as significant.

### Additional resources

• More information about the clinical trials included in this manuscript can be found on ClinicalTrials.gov:
  ○ Congenital Heart Disease GEnetic NEtwork Study (CHD GENES), ID NCT01196182.
  ○ Genomic Basis of Neurodevelopmental and Brain Outcomes in Congenital Heart Disease (CHD Brain and Genes), ID NCT03070197.

## Supplemental information

Document S1. Figures S1–S7

Table S1. Results from two-sided Mann-Whitney U-test for MACIE scores

Table S2. List of 5,313 epigenetic annotations

Table S3. List of 583 heart/brain annotations

Table S4. Modules resulted from WGCNA with the negative scores and their corresponding clustered annotations

Table S5. Sulcal pattern-eigen module correlations along with its *p*-values of the WGCNA with the negative scores

Table S6. Partial correlation between functional scores and sulcal pattern traits while controlling for potential confounders

Table S7. Modules resulted from WGCNA with the positive scores and their corresponding clustered annotations

Table S8. Sulcal pattern-eigen module correlations along with its *p*-values of the WGCNA with the positive scores

Table S9. Results from two-sided Mann-Whitney U-test between scores from the subset of 30 participants with MRI and the rest of the cohort

Table S10. Results from one-sided Mann-Whitney U-test for negative and positive scores predicted by Basenji2

Table S11. Results from one-sided Mann-Whitney U-test for negative and positive scores predicted by Enformer

Table S12. Results from two-sided independent t-test between the subset of participants with genotype and phenotype data (n = 30) and the rest of the participants with phenotype data (n = 62)

Table S13. Results from linear regression models predicting for sulcal pattern traits of the case (n = 92) and control (n = 94) groups

Table S14. Output from enrichment analysis

Table S15. Genes enriched along with the number of GO terms that clustered it, the number of ncDNVs that multi-hit that gene, and the SNPs

Table S16. ncDNVs that overlap with ENCODE Candidate Cis-Regulatory Elements along with the gene that they multi-hit

Data S1. Sulcal pattern similarity values, related to Figures 2C, 3A, and 3B
