# Supplementary material for: Noncoding variants and sulcal patterns in congenital heart disease: Machine learning to predict functional impact
Source: iScience. 2024 Dec 28;28(2):111707. doi: 10.1016/j.isci.2024.111707 (PMC11772982; doi:10.1016/j.isci.2024.111707)
Supplement: Document S1. Figures S1–S7 [file mmc1.pdf]

iScience, Volume *28*

Supplemental information

Noncoding variants and sulcal patterns in congenital heart disease: Machine learning to predict functional impact

Enrique Mondragon-Estrada, Jane W. Newburger, Steven R. DePalma, Martina Brueckner, John Cleveland, Wendy K. Chung, Bruce D. Gelb, Elizabeth Goldmuntz, Donald J. Hagler Jr., Hao Huang, Patrick McQuillen, Thomas A. Miller, Ashok Panigrahy, George A. Porter Jr., Amy E. Roberts, Caitlin K. Rollins, Mark W. Russell, Martin Tristani-Firouzi, P. Ellen Grant, Kiho Im, and Sarah U. Morton

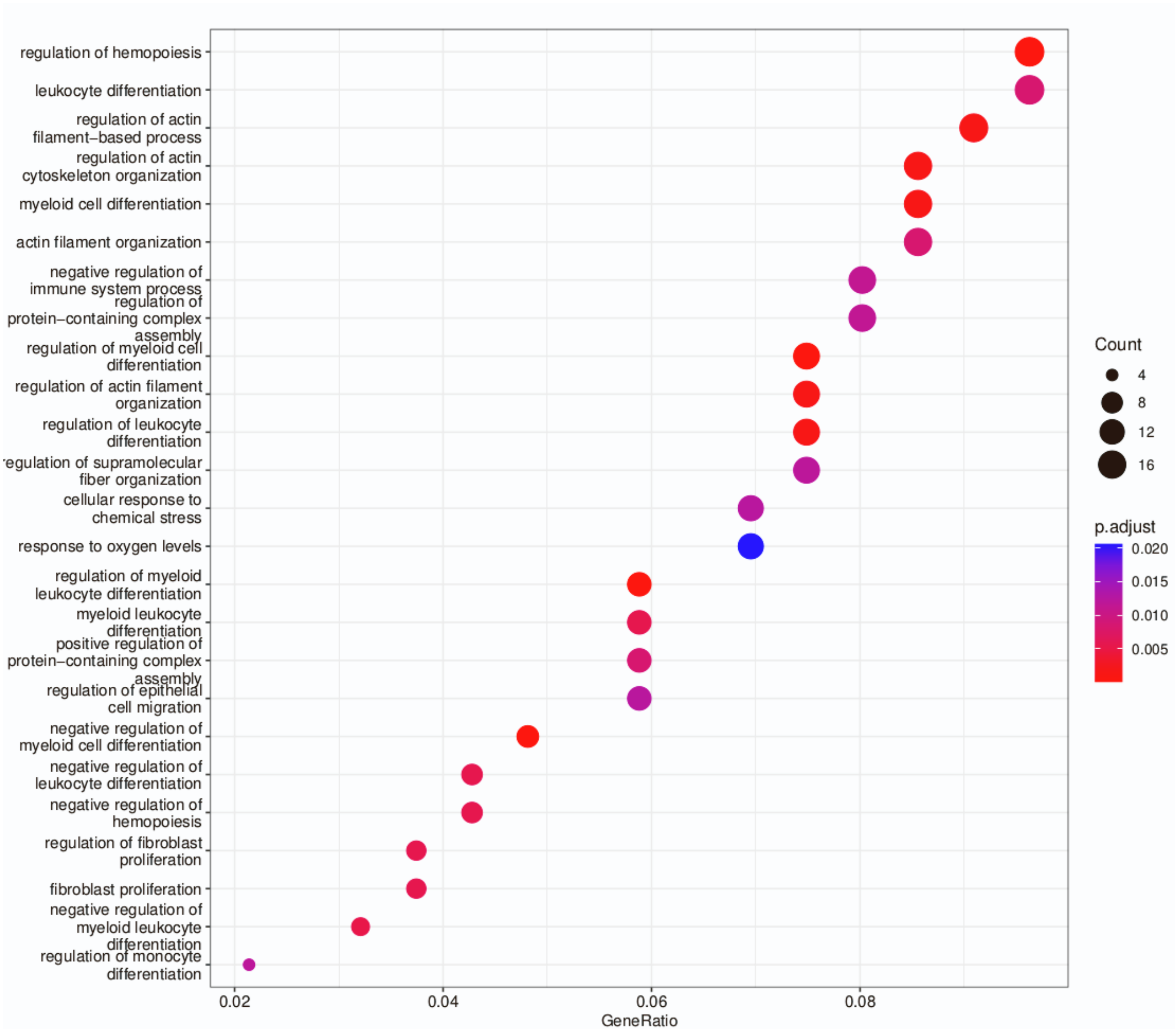

Figure S1. Functional enrichment of highest scored variants estimated to be regulatory in CHD by MACIE, Related to STAR Methods. Highest scoring variants in the regulatory category from MACIE were different between the CHD and non-CHD groups. Abbreviations: CHD, congenital heart disease.

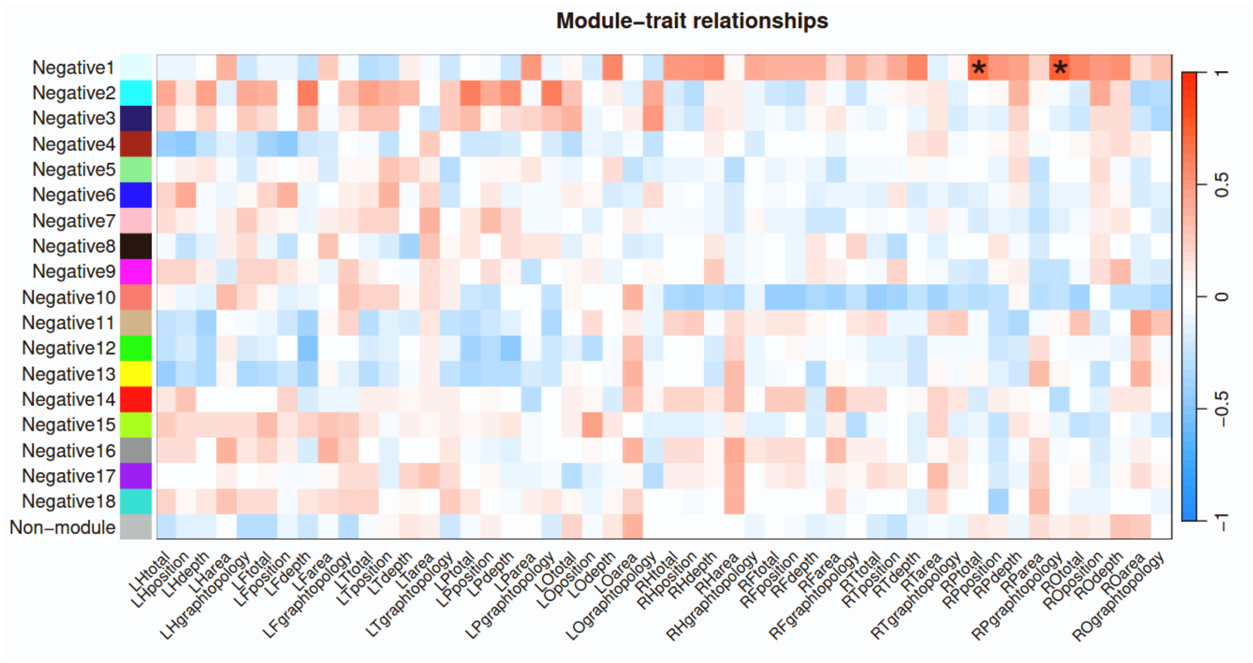

Figure S2. Heatmap with modules-sulcal pattern coefficient correlations and Student asymptotic p-values of the network computed with the minimum scores, Related to STAR Methods. An asterisk indicates a significant correlation using Bonferroni criteria. Bonferroni p-value threshold was based on the 950 module-sulcal feature correlations (α = 0.05, n = 950, P = 5.26e-05). Abbreviations: L, Left; R, Right; F, frontal; H, hemisphere; O, occipital; P, parietal; T, temporal.

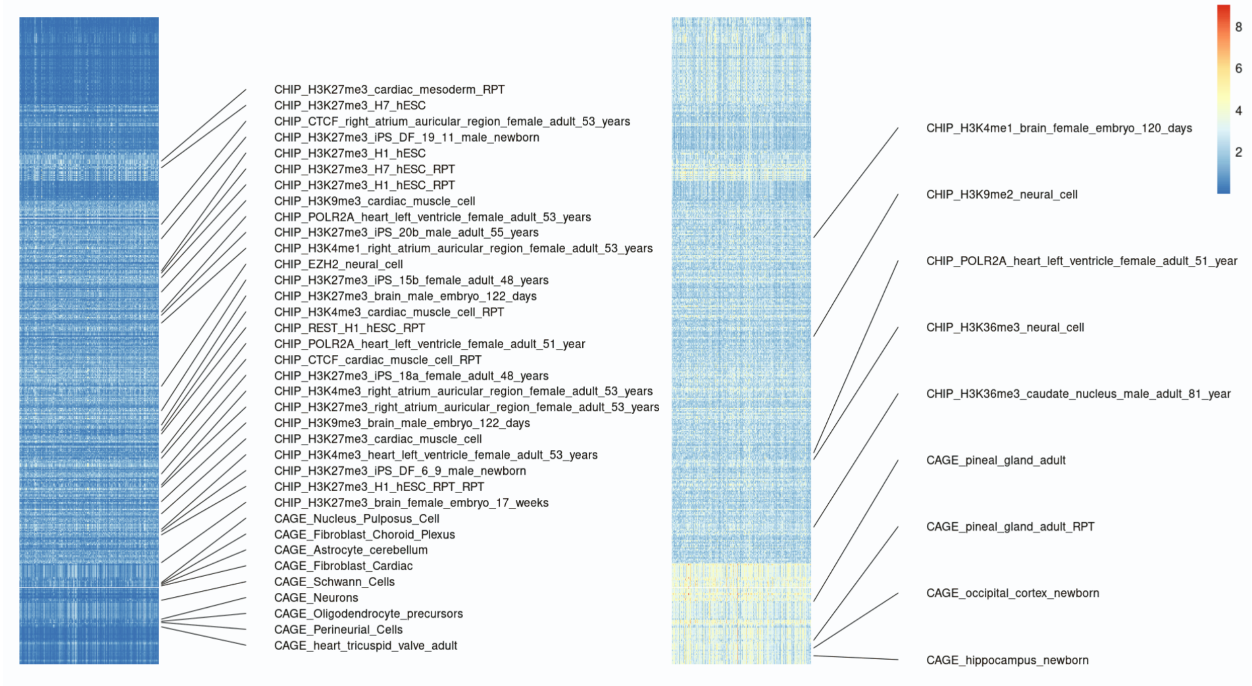

Figure S3. Heatmaps with positive functional scores predicted by Basenji2 (left) and Enformer (right), Related to STAR Methods. Comparison of the highest positive functional prediction per annotation (rows) for each participant with CHD (columns). The names of the annotations that were different between CHD and non-CHD are displayed. Functional scores are log-transformed [log(x + 1)] for better visualization. Abbreviations: CHD, congenital heart disease.

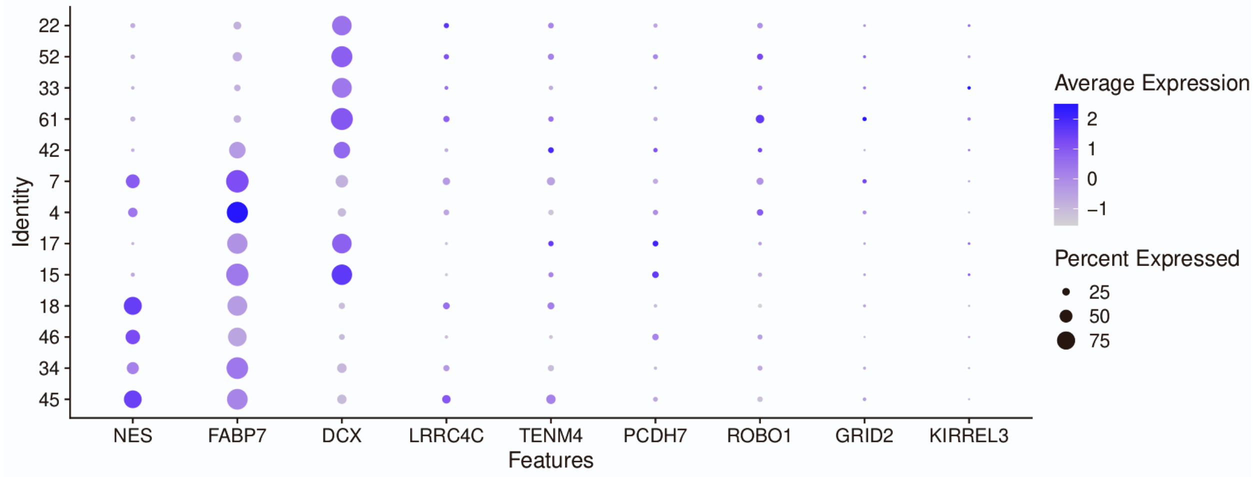

Figure S4. Feature expression changes across different radial glial clusters in the early brain, Related to STAR Methods. Dot plot visualization of the percentage and average expression of genes predicted to be regulated by ncDNVs and different radial glial clusters in early brain development. *NES*, *FABP7* and *DCX* are standard markers for stages of neural progenitor development and support the conclusion that *LRRC4C*, *TENM4* and *PCDH7* are expressed earlier in neuronal development than *ROBO1*, *GRID2* and *KIRREL3*. Abbreviations: ncDNVs, noncoding *de novo* variants.

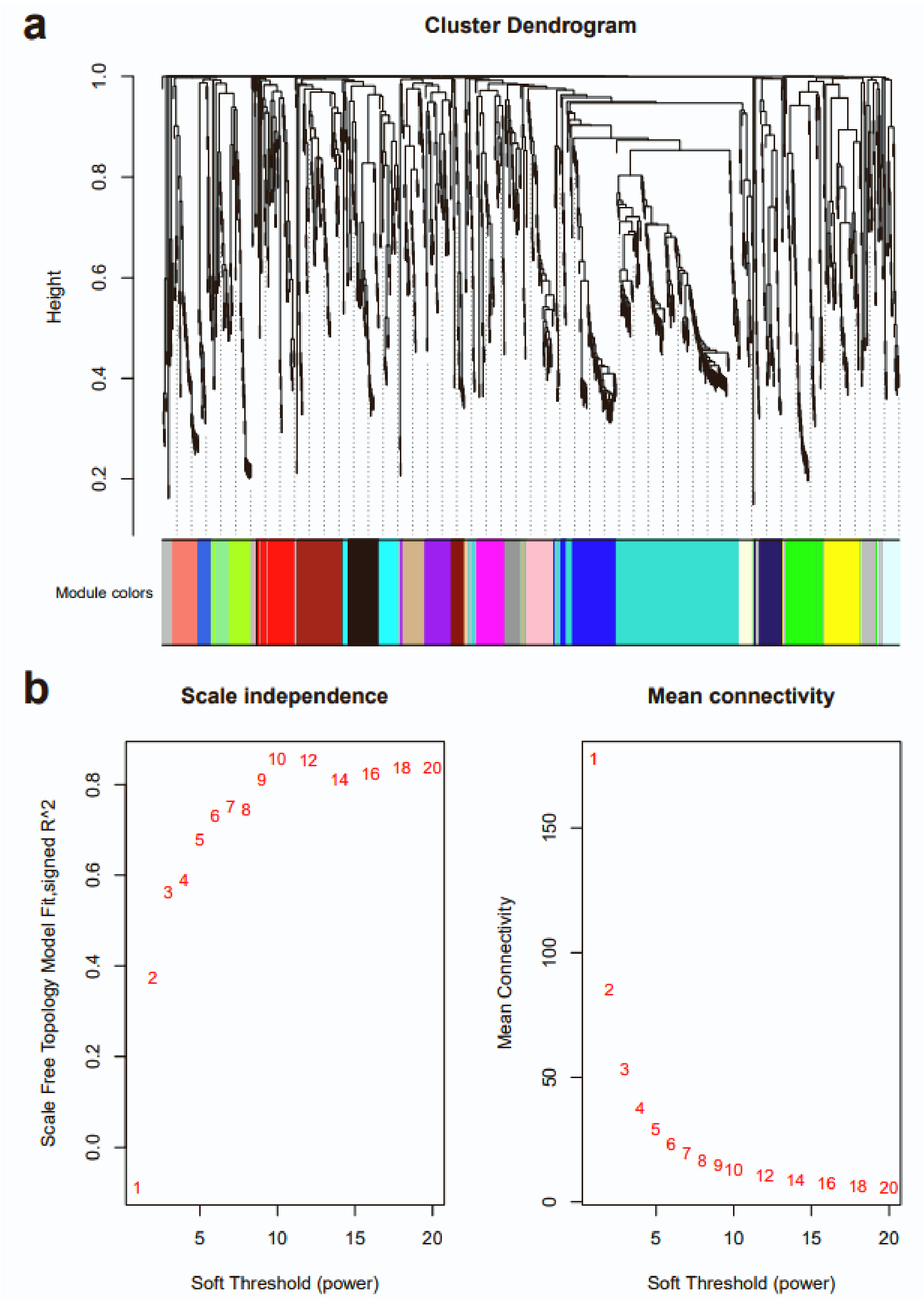

Figure S5. a, Hierarchical clustering dendrogram and modules detected in the constructed signed hybrid network with the positive scores, Related to STAR Methods. b, Scale-free topology model fit for a signed hybrid network built with the maximum functional scores (left) and mean connectivity (right). Different networks were computed using different soft thresholding powers and a final version with β=9 was selected.

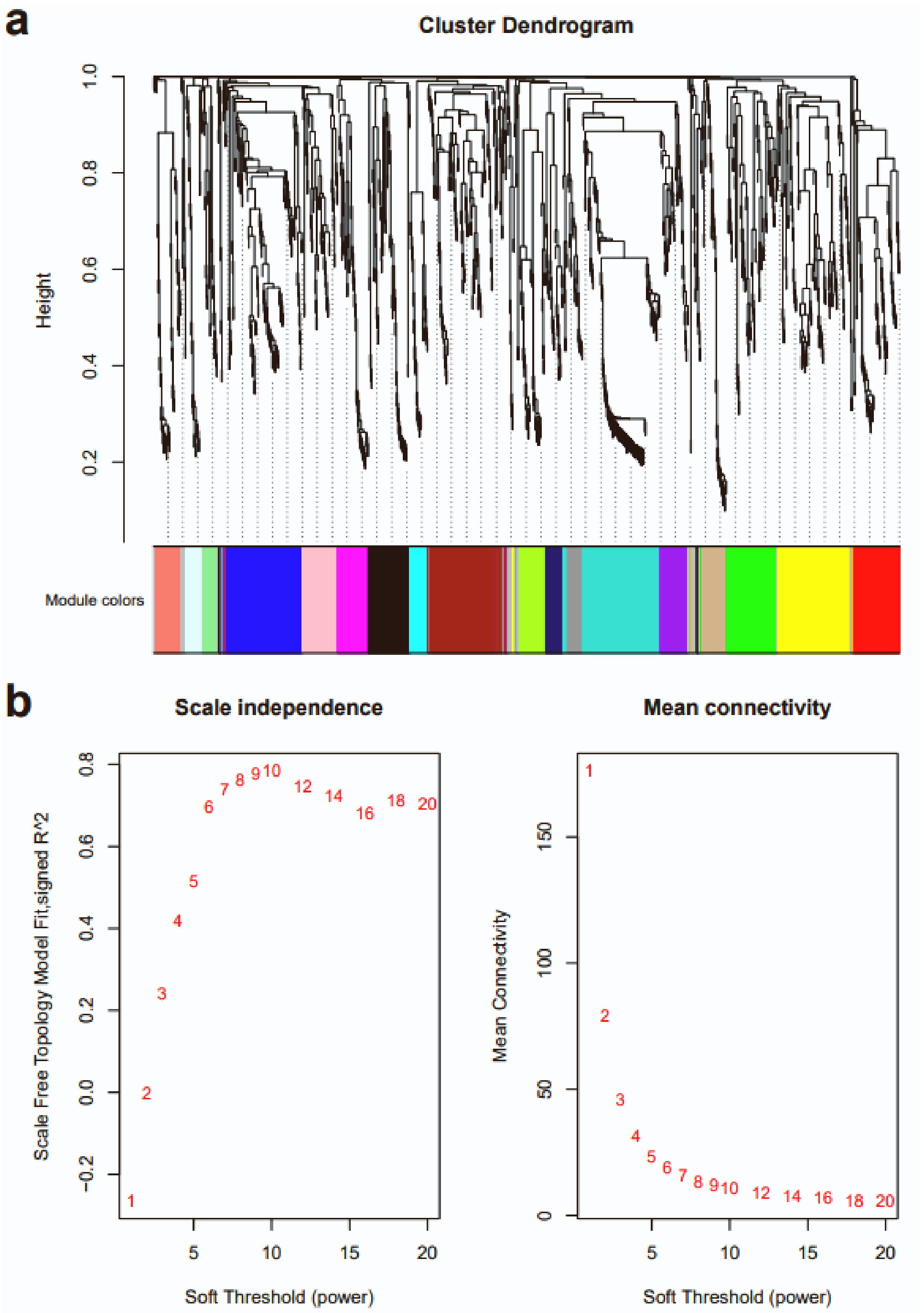

Figure S6. a, Hierarchical clustering dendrogram and modules detected in the constructed signed hybrid network with the negative scores, related to STAR Methods. b, Scale free topology model fit for a signed hybrid network built with the minimum functional scores (left) and mean connectivity (right). Different networks were computed using different soft thresholding powers and a final version with β = 9 was selected.

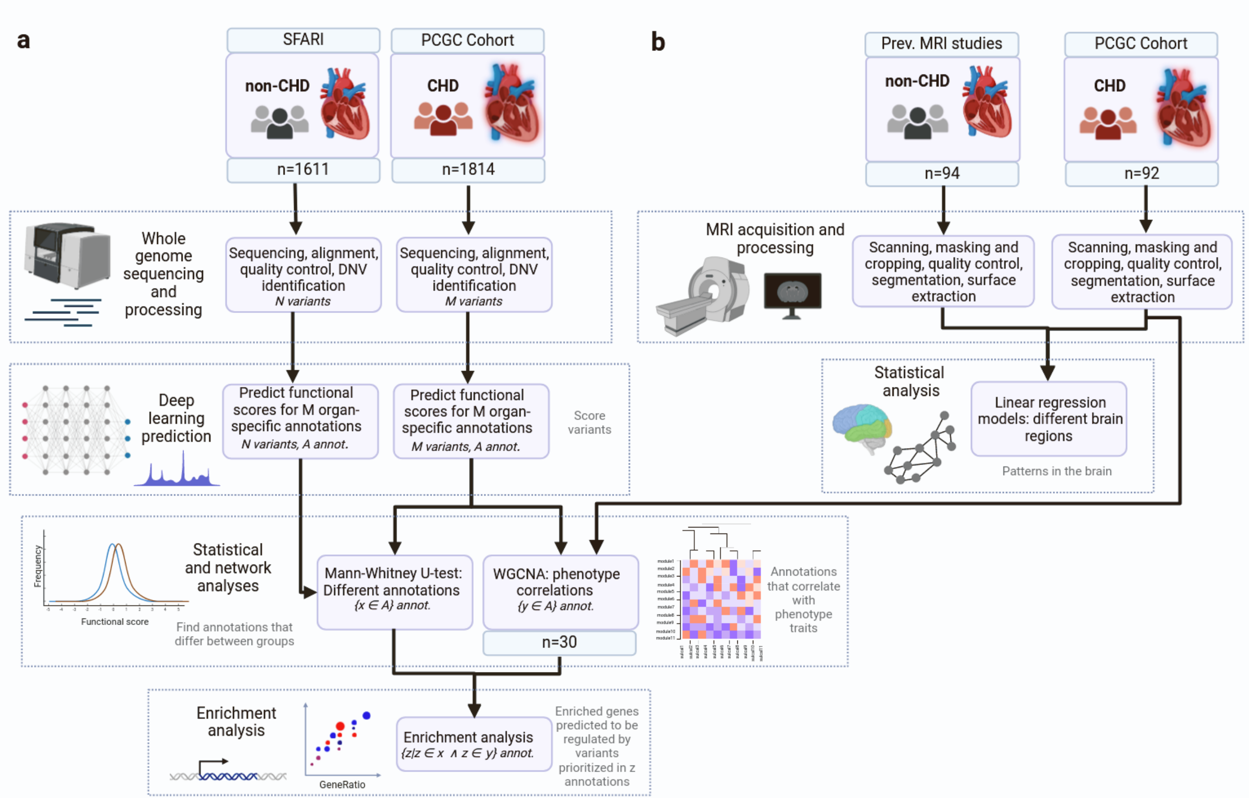

Figure S7. a, Analysis workflow followed to explore, analyse, correlate, and prioritise CHD's ncDNVs based on the functional scores predicted by Basenji2 and Enformer in 583 heart/brain annotations, related to STAR Methods. One-sided Mann-Whitney U-tests were performed using the negative and positive scores to seek differences between the CHD and non-CHD ncDNVs. Parallel, WGCNA was carried out to find correlations between CHD's ncDNVs and their sulcal pattern traits. One annotation (H3K9me2-neural) was prioritised by both previous steps, whose ncDNVs were analysed using enrichment analysis. b, Analysis workflow used to explore sulcal pattern similarity coefficient in the CHD (n = 92) and non-CHD (n = 94) groups. Linear regression models were computed for each of the 50 measurements to evaluate the extent to which CHD status (Category 0: non-CHD, Category 1: CHD status) affects sulcal measurements. Abbreviations: CHD, congenital heart disease; ncDNVs, noncoding *de novo* variants.
